# Pharmacotherapy and Stone Mineral Subtype Influence Long-Term Recurrence Rates in Calcium Stone Formers

**DOI:** 10.34067/KID.0000000000000526

**Published:** 2024-07-25

**Authors:** Rupam Ruchi, Elizabeth A. Di Valerio, Shahab Bozorgmehri, Michael Waseer Bacchus, Benjamin K. Canales, Russell Terry, John Michael DiBianco, Vincent G. Bird

**Affiliations:** 1 Division of Nephrology, Hypertension, and Renal Transplantation, University of Florida, Gainesville, FL; 2 Department of Urology, University of Florida, Gainesville, FL

**Keywords:** epidemiology and outcomes, kidney stones

## Abstract

**Key Points:**

- Long-term recurrence data on pure metabolic calcium stone formers are limited.
- The presented data highlight the importance of medical therapy in preventing future stones among high-risk patients.
- Our study shows that the benefit of medical therapy may take 5 years to be evident; this fact should be considered in planning future studies.

**Background:**

Long-term recurrence data on kidney stones are limited. We investigated stone recurrence in calcium-oxalate (CaOx) and calcium-phosphate (CaP) stone formers over a 10- to 12-year follow-up period.

**Methods:**

We retrospectively identified patients from a surgical database with (*1*) CaOx or CaP stones, (*2*) postsurgical computed tomography imaging, and (*3*) at least 10 years of clinical follow-up and imaging. Data on medical therapy (MT), defined as being on thiazide/thiazide-like diuretic, potassium citrate, and/or allopurinol, were collected. Patients' records were reviewed for stone recurrence over a 10- to 12-year period. Associations between stone type, MT, and time to recurrence were analyzed with Kaplan–Meier survival curves and Cox proportional hazard models. Multivariate analysis was done using the Cox proportional hazard model.

**Results:**

Of the 149 individuals who met inclusion criteria, 87 (58.3%) underwent baseline 24-hour urine testing, and 46 (30.8%) were prescribed MT in the form of thiazide (26/46; 57%), potassium citrate (25/46; 54%), and allopurinol (5/46; 11%). Compared with non-MT patients, patients on MT were more likely to have diagnosis of hypertension (*P* = 0.008) and be hypocitraturic at baseline (*P* = 0.01). Over a mean of 10.6 years, patients on MT had significantly fewer stone events compared with those not on MT (21.3% versus 37.5%, *P* = 0.04), with 8 (17%) individuals discontinuing their MT over the study period. Patients with predominantly CaP mineral subtype had more stone events than CaOx (64% versus 36%, *P* = 0.006), a phenomenon likely driven by higher baseline urine pH (>6, 58.8% versus 33.9%, *P* = 0.02). By survival analysis, the impact of stone subtype and MT became apparent at follow-up months 20 and 60, respectively.

**Conclusions:**

In a population of calcium stone formers at high recurrence risk, patients with CaOx mineral subtype and on MT had the lowest stone event rate on long-term follow-up. These findings suggest that the beneficial effect of MT may take up to 5 years to become evident clinically and by surveillance imaging.

## Visual Abstract

**Figure:**
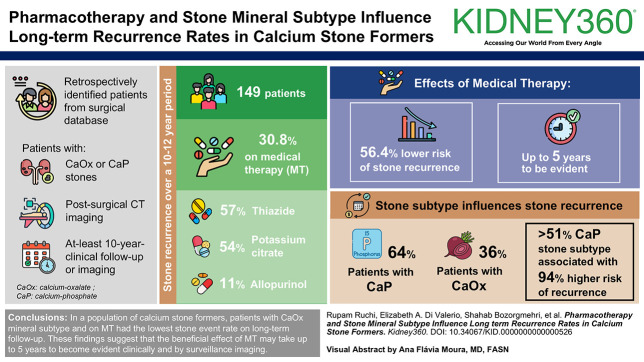

## Introduction

The prevalence of kidney stones has increased in industrialized nations, with reported rates of 3.2% in 1980, 8.8% in 2010, and 9.7% in 2018.^1,2^ The economic burden has correspondingly increased with approximately $3 billion in estimated US expenditures in 2000^3^ to approximately $10 billion in 2006,^4^ most of which were related to surgical procedures. Finally, kidney stone disease has a high recurrence rate, with up to 60% of patients experiencing one recurrence and up to 20% recurring at least four times throughout their lifetime.^4^ In light of increasing prevalence, recurrence, and economic burden, there is dire need for management strategies to stratify stone risk and prevent recurrence.^5^

The majority of kidney stones contain a heterogeneous mix of calcium oxalate (CaOx) and calcium-phosphate (CaP) mineral. Characterizing stone formers by their predominant mineral subtype can help clinicians identify underlying causes and stratify patient stone risk.^6^ Despite much previous work on the effectiveness of medical therapy (MT) for short-term prevention,^3^ only a handful of studies have attempted to characterize long-term recurrence risk in noninfectious calcium stone formers. Thus, the objective of this study was to characterize patients with CaOx or CaP (noninfectious) kidney stones and investigate the impact of MT on clinical outcomes over a prolonged surveillance period.

## Methods

Using an Institutional Review Board–approved dataset that includes all patients at our tertiary hospital who underwent a kidney stone procedure, we identified patients older than 18 years who underwent either ureteroscopy (URS) or percutaneous stone removal over a 2-year period (July 28, 2010, to July 27, 2012). The surgical intent in these patients was removal of all luminal stones. The 2010 date was chosen as this was the inception of electronic medical record (electronic medical record [EMR], Epic Systems; Verona, WI) within our institution. From that subset, we identified patients with CaOx (any subtype) or CaP (hydroxyapatite and brushite) stone mineral analysis (Louis C. Herring and Co, Orlando, FL). We included only patients who had postoperative imaging within 6–12 weeks of their procedure (ultrasound or CT as baseline study) and had ten or more years of annual follow-up visits and (at least) every 12- to 18-month imaging within our metabolic stone clinic. Stone-free status was determined at the time of surgery by endoscopy and confirmed by postoperative imaging. We excluded patients with other mineral subtypes in their stone specimen (uric acid, carbonate apatite, struvite, cystine) and known medical conditions associated with stones, including previous bariatric surgery, chronic diarrheal states, sarcoidosis, primary hyperparathyroidism, and medullary sponge kidney. As standard in our clinic, all patients were offered baseline 24-hour urine testing (Litholink, Chicago, IL) and had clinic follow-up visits to discuss the results and general stone prevention strategies, such as increasing oral fluid intake and limiting dietary sodium. A diet low in oxalate was recommended for patients with any oxalate stone content. MT (thiazide/thiazide like diuretics, citrate, allopurinol as appropriate) was offered to all patients within first 6 months as they were considered high risk because of their recent procedure. Patients who were started on MT outside of this 6-month baseline visit were excluded from this study.

The data extracted from patient charts included baseline demographics, comorbidities, stone passage history, previous stone interventions (before this operative procedure), stone type, and metabolic studies (baseline and follow-up 24-hour urine and serum testing when available). As kidney stone disease is more common among the White population, we included self-reported race in our initial demographic screening. Stone formers were grouped by primary mineral subtype CaOx (content ≥51%) or CaP (content ≥51%). Data on MT, defined as being on thiazide or thiazide-like diuretic, alkaline therapy, and/or allopurinol, were also collected. We classified individuals as on MT if they were on any of these medications at the time of enrollment or were started on MT within 6 months of their procedure. Medication adherence was verified by reviewing clinical notes and by refill documentation through hospital or pharmacy EMR. Patients were considered not on MT (non-MT) if they declined medication prescriptions or if they failed to fill their prescriptions at the pharmacy. Two reviewers, blinded to each other's findings, recorded all baseline clinical variables and imaging results, including stone number, size, and location (if stone present). When the reviewers' findings differed, an arbitrator (R. Ruchi, board-certified nephrologist) served as a third reviewer. Patients were followed until July 28, 2022, or until a stone event occurred, defined as (*1*) presence of new stone on imaging compared to baseline, (*2*) stone growth >2 mm, (*3*) stone passage (confirmed by imaging and/or collected stone), or (*4*) kidney stone procedure.

Baseline demographic and clinical characteristics were compared between groups using the t-test or Wilcoxon rank-sum test for normally and non-normally distributed continuous variables and the chi-squared test for categorical variables. We determined the association between stone type (CaOx, CaP) and therapy (MT, no MT) and time to stone recurrence using Kaplan–Meier (KM) survival curves, log-rank test, and Cox proportional hazard models. Multivariate analysis was performed using the Cox proportional hazard model. The independent variables included in the multivariate model are stone type, MT, number of stone events, hypocitraturia (defined as 24-hour urine citrate <550 mg in women and <450 mg in men), hypercalciuria (defined as 24-hour urine calcium >200 mg in women and >250 mg in men), and urine pH > 6. The dependent variable is time to stone recurrence. Data are presented as hazard ratios (HRs) with 95% confidence intervals (CIs). All significance tests were two-sided, with a *P* < 0.05 considered statistically significant. Statistical analyses were performed using Statistical Analysis Software version 9.4 (SAS Institute Inc., Cary, NC).

## Results

We identified 284 patients who had procedures within study timeframe and continued to have active EMR activity at our institution for at least 10 years. Of them, 68 were excluded for no stone analysis (*N*=5), noncalcium stone composition (*N*=56), or known predisposing medical conditions (*N*=7). In addition, 67 patients were excluded because they did not have at least q18 month imaging in our system (*N*=14) or were lost to follow-up (*N*=53). Ultimately, 149 patients met inclusion criteria and were divided into CaOx or CaP subtypes and into MT or no MT.

Baseline demographic and total group characteristics are shown in Table 1 and further stratified by MT and no MT. Most patients were White (87%), middle-aged (mean 50.5 ± 15 years), and had equally distributed sex. At least one kidney stone surgical intervention before study procedure was reported by 105 individuals (70.5%), including previous shock wave lithotripsy (*n*=41; 27.5%), URS (*n*=112; 75.2%), and/or percutaneous nephrolithotomy (*n*=57; 38.3%). Demographics between groups were similar except MT were more likely to have a diagnosis of hypertension (76.1% versus 53.4%, *P* = 0.008) compared with no MT. Those on MT also reported higher rates of previous shock wave lithotripsy (41.3% versus 21.3%, *P* = 0.0118) and percutaneous nephrolithotomy (54.3% versus 31.1, *P* = 0.006) than in the no MT group with no difference in previous URS. CaOx and CaP stone subtypes were also similar between groups. Of the entire cohort, 87 (58.3%) patients underwent a baseline 24-hour urine study within 6 months of their procedure (Table 2). Most common abnormalities were high urine sodium (>100 mmol, *n*=69, 79.3%) and low urine volumes (<2000 ml, *n*=64, 73.6%). Urine parameters were not significantly different between the two groups except patients on MT had higher rates of hypocitraturia (71.9% versus 45.5%, *P* = 0.01) compared with no MT group. Follow-up urine studies were available only for 24 (16.1%) patients. This small number was insufficient for meaningful statistical analysis.

**Table 1:** Demographics and clinical characteristics of study cohorts

Variable	MT (*n*=46)	No MT (*n*=103)	Total (*n*=149)	*P* Value
Age, yr	53.5 ± 14.9	49.2 ± 15.3	50.5 ± 15.3	0.12
BMI (kg/m^2^)	32.5 ± 8.7	30.6 ± 7.9	31.2 ± 2.6	0.20
No. of past stone passages/procedures	3 ± 2.1	2.7 ± 2.8	2.8 ± 2.6	0.41
Sex (% male)	24 (52%)	49 (48%)	73 (49%)	0.6
Self-reported race (White)	39 (84.8%)	91 (88.3%)	130 (87%)	0.5
Hypertension	35 (76.1%)	55 (53.4%)	90 (60.4%)	0.008
Diabetes	15 (32.6%)	24 (23.3%)	39 (26.1%)	0.23
Hyperlipidemia	21 (45.6%)	32 (31.1%)	53 (35.5%)	0.08
CAD	7 (15.2%)	15 (14.6%)	22 (14.8%)	0.91
CKD	4 (8.7%)	9 (8.7%)	13 (8.7%)	0.99
Recurrent UTI	14 (30.4%)	20 (19.4%)	34 (22.8%)	0.14
≥51% CaP	4 (8.7%)	21 (20.4%)	25 (16.8%)	0.07

All means are shown ± SD. BMI, body mass index; CAD, coronary artery disease; CaOx, primarily, ≥51% calcium oxalate mineral content; CaP, ≥51% calcium phosphate mineral content; CKD, CKD defined as an eGFR <60 ml/min per 1.73 m^2^; MT, medical therapy; UTI, urinary tract infection.

**Table 2:** Baseline 24-hour urine parameters in patients with and without medical therapy

24-h Urine Variable, Mean ± SD	MT (*n*=32)	No MT (*n*=55)	Total (*n*=87)	*P* Value
Calcium (mg/d)	195 ± 116	232 ± 120	219 ± 119	0.1
Citrate (mg/d)	382 ± 303	576 ± 307	506 ± 318	0.002
Oxalate (mg/d)	37.2 ± 14.1	36.1 ± 15.9	36.4 ± 15.2	0.663
Uric acid (g/d)	0.56 ± 0.25	0.64 ± 0.24	0.61 ± 0.24	0.083
Sodium (mmol/d)	154 ± 70	162 ± 81	159 ± 77	0.891
Potassium (mmol/d)	55.1 ± 29.5	55.9 ± 22.4	55.6 ± 25.0	0.591
Ammonium (mmol/d)	40.2 ± 49.4	33.3 ± 14.9	35.8 ± 32.0	0.819
pH	5.99 ± 0.56	5.97 ± 0.49	5.97 ± 0.51	0.596
Urine volume (ml/d)	1780 ± 953	1627 ± 722	1682 ± 809	0.578
SS CaOx	7.52 ± 4.045	8.50 ± 3.40	8.15 ± 3.65	0.12
SS brushite	1.32 ± 0.95	1.51 ± 1.05	1.44 ± 1.01	0.462
SS uric acid	0.87 ± 0.77	1.21 ± 0.99	1.08 ± 0.93	0.138

CaOx, calcium-oxalate; MT, medical therapy; SS, supersaturation.

Within 6 months of their study procedure, 46 (30.8%) patients were either started on (*n*=28) or asked to remain on (*n*=18) MT, including 26 (56.5%) on thiazides, 25 (54.3%) on potassium citrate, and 5 (10.9%) on allopurinol. We found that hydrochlorothiazide (HCTZ) was the only thiazide diuretic used by our patients. None of the patients were taking thiazide-like diuretics. Ten patients (21.7%) were on combination pharmaceuticals. A summary of MT in relation to the time of initiation and prescription indication is shown in Table 3. Over the ensuing 10 years, eight patients (17.4%) discontinued MT, including five patients on thiazide (three because of hypokalemia, one because of orthostatic hypotension, and one because of hyperglycemia) and three on potassium citrate (two because of abdominal discomfort and muscle weakness and one because of cost).

**Table 3:** Summary of pharmacotherapy agents used during study (*n*=46)

Medications	Taking before Inclusion in Study, Prescribed Empirically	Taking before Inclusion in Study, Prescribed Based on Prior 24-h Urine Study	Started Empirically at First Postoperative Visit	Started Based on 24-h Urine Obtained after First Postoperative Visit	Total
Potassium (K) citrate	2	4	—	11	17
HCTZ	5^a^	5^b^	7	1^c^	18
Allopurinol	—	—	1^d^	—	1
K citrate and HCTZ	—	—	3^e^	3	6
K citrate and allopurinol	—	1^f^	1^g^	—	2
HCTZ and allopurinol	2^h^	—	—	—	2
Total	9	10	12	15	46

HCTZ, hydrochlorothiazide.

a Four monotherapy for hypertension, one lisinopril/hydrochlorothiazide.

b Four monotherapy for hypercalciuria and hypertension, one losartan/hydrochlorothiazide.

c Hydrochlorothiazide dose increased from 25 mg once daily to 25 mg twice daily for persistent hypercalciuria.

d 300 mg once daily for high urine uric acid (>0.75 g in women and >0.8 g in men).

e K citrate added to hydrochlorothiazide.

f Potassium citrate added for hypocitraturia.

g Allopurinol for high urine and serum uric acid.

h Treatment for hypertension and high serum uric acid.

Patients were then grouped as stone event (new stone, growth, passage, or procedure) or no stone event. Overall, 61 (40.9%) patients had stone event over mean period of 10.6 ± 0.9 years. Groups were similar in baseline factors, rates of MT discontinuation, and comorbidities except a higher number of patients in the stone event group (*n*=52, 85.2%) had ≥2 previous surgical interventions compared with no stone event (*n*=53, 60.2%; *P* = 0.002). Stone event patients were more likely to have CaP stone mineral content (*n*=16, 26.2%) than no stone event (*n*=9, 10.2%; *P* = 0.006) and higher baseline urine pH > 6 (58.8% versus 33.9%, *P* = 0.02). Stone event patients were less likely to be on MT compared with no stone event (21.3% versus 37.5%, *P* = 0.04). By univariate analysis, MT reduced the risk of stone event rate by 47.3% (HR, 0.527; 95% CI, 0.285 to 0.975). By multivariate analysis, patients on MT had a 56.4% lower risk of stone recurrence than those not on MT (HR, 0.436; 95% CI, 0.230 to 0.824). In addition, ≥51% CaP content (HR, 1.941; 95% CI, 1.059 to 3.557), number of past stone events (HR, 1.155; 95% CI, 1.077 to 1.238), and urine pH > 6 (HR, 2.096; 95% CI, 1.184 to 3.709) remained statistically significant (Table 4). However, 24-hour urine calcium analyzed as a continuous variable was notably not significantly different between the two groups (220.4 ± 81.0 mg in the stone event group versus 218.2 ± 139.8 in the no stone event group, *P* = 0.55).

**Table 4:** Association between medical therapy and stone event by multivariate Cox proportional hazard model

Independent Variable	HR (95% CI)	*P* Value
MT^a^	0.436 (0.230 to 0.824)	0.017
≥51% CaP mineral content	1.941 (1.059 to 3.557)	0.031
No. of past stone passages/procedures	1.155 (1.077 to 1.238)	<0.001
Hypocitraturia^b^	1.286 (0.695 to 2.379)	0.422
Hypercalciuria^c^	1.134 (0.599 to 2.146)	0.699
Urine pH > 6	2.096 (1.184 to 3.709)	0.011

CaP, calcium-phosphate; CI, confidence interval; HR, hazard ratio; MT, medical therapy.

a Medical therapy includes use of thiazide, allopurinol, and alkaline therapies.

b Hypocitraturia defined as citrate <450 mg in men and <550 mg in women.

c Hypercalciuria defined as calcium >250 mg in men and >200 mg in women.

**Figure 1:**
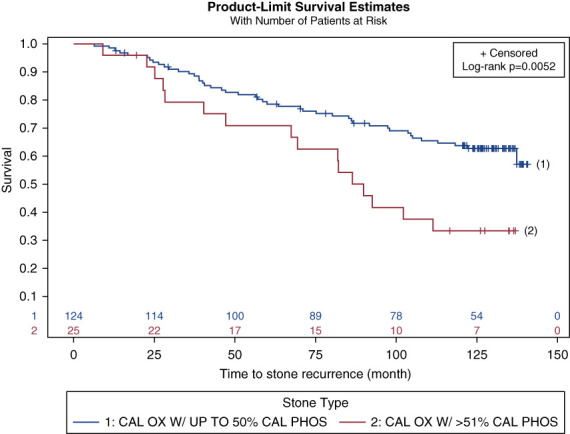
**KM curves for stone recurrence according to stone subtype.** Patients with <50% CaP stone mineral content (blue line, labeled 1) show a lower recurrence rate than patients with a higher CaP content (red line, labeled 2), with the curves diverging at around 20 months of postoperative surveillance. CaOx, calcium-oxalate; CaP, calcium-phosphate; KM, Kaplan–Meier.

**Figure 2:**
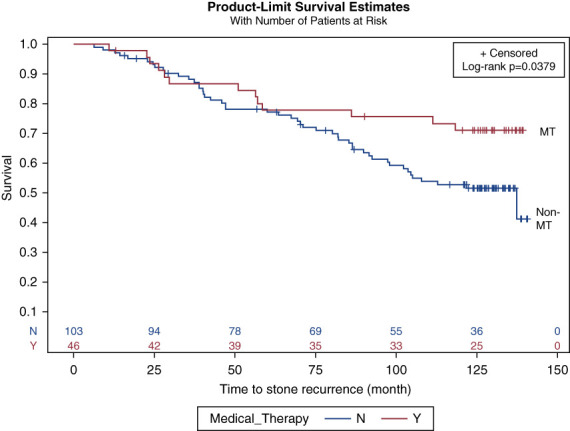
**KM curves for stone recurrence according to MT.** Patients on MT (red line) show a lower recurrence rate than those not on MT (blue line), with the curves diverging at around 60 months of postoperative surveillance. MT, medical therapy.

**Figure 3:**
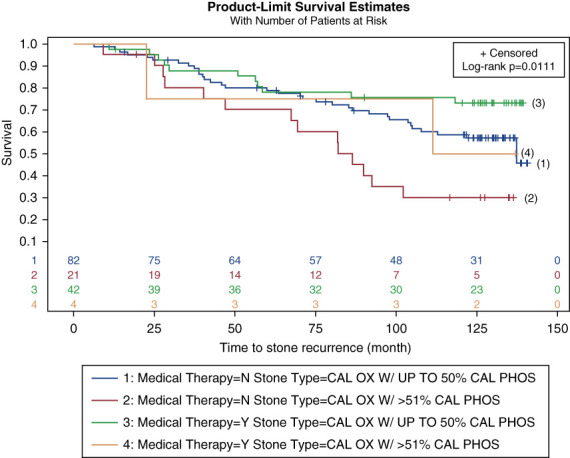
**KM curves for stone recurrence according to stone type and pharmaceutical intervention.** Patients who received MT and had <50% CaP stone subtype [represented by the green line, labeled (3)] had the lowest recurrence rate. Patients with >51% CaP stone subtype and not on MT [depicted by the red line, labeled (2)] had the highest recurrence rate. The blue line [labeled (1)] indicates patients not on MT and <50% CaP mineral content; the brown line [labeled (4)] depicts patients on MT and higher CaP stone mineral content.

Survival curves were generated for stone recurrence according to the CaP mineral content and MT (Figures 1 and 2). Patients with ≥51% CaP stone subtype were at 94% higher risk of recurrent stones than CaOx. Figure 3 shows the KM curves for stone recurrence according to stone subtype and MT combined, and patients with ≥51% CaP stone subtype and no MT were at the highest risk of recurrence. We identified that the separation of the KM curves in Figure 2 does not occur until at least after 60 months, implying that the benefit of MT on stone recurrence is not evident until after at least 5 years of initial enrollment when they had their last procedure for stones. Similarly, the effect of stone subtype is not evident until at least 20 months after the last procedure.

## Discussion

Kidney stone recurrence is reported in 35%–50% patients without specific treatment.^7^ Factors such as history and number of previous stone events have been associated with higher rates.^8^ Our study shows a high recurrence rate of 40.9% over a period of 10–12 years. All patients in our study had a stone event requiring a procedure at enrollment. The majority of patients in our group had experienced stone events before the index procedure, with an average of 2.8±2.6 stone events in the past, indicating that they were already at high risk. Therefore, the high recurrence rate in our study aligns with findings from previous studies.

Despite being at high risk for recurrence, only 30.8% of our patients were on MT. The most common class of medical treatment was thiazides, followed by citrate. Significantly higher percentage of patients receiving MT had history of hypertension. Because thiazides are a common first-line drug for its treatment, it is possible that the use of thiazides in these patients was driven by their history of hypertension.

Twenty-four-hour urine testing is currently recommended by both American and European Urological Associations as a standard workup for patients with recurrent kidney stones. However, in practice, it is not universally done. Patients may find it cumbersome, insurance companies may not cover it, and all physicians may not be well versed in interpreting these tests.^9^ One study found an overall 24-hour urinalysis rate of 7.4% among patients at high risk of recurrence. In another study of 130,489 veterans with urinary stone disease, only 13% had completed a 24-hour urine testing.^10^ While 24-hour urine analysis was offered to all patients at the time of enrollment in our study, it was only completed by 58.3% of patients. While this rate is much higher than previously reported rates, this also highlights the difficulties of obtaining a 24-hour urine collection even in patients who have required recent procedures for kidney stones and perhaps are most motivated. Interestingly, the only significant difference in 24-hour urine parameters among MT versus non-MT was low urinary citrate, which seems to have been the driving force for use of citrate therapy. The 24-hour urine calcium was not significantly different between the two groups.

The primary outcome of our study was to examine the long-term recurrence of calcium-containing metabolic kidney stones over a 10- to 12-year follow-up period. Very few studies have reported long-term recurrence data on kidney stones in recent literature. In 2009, Parks and Coe^11^ analyzed long-term outcomes among patients with various types of kidney stones, including uric acid, struvite, and cystine, as well as patients with bowel disease and primary hyperparathyroidism. As reported by Andrew Rule and colleagues, stone composition plays an important role in determining recurrence, with the highest rate for brushite, struvite, and uric acid stones. Cystine stones have a very high recurrence rate as well.^12^ By excluding these patients, our study is able to examine the long-term outcome of pure metabolic calcium-containing kidney stones. Similarly, Singh and colleagues^13^ conducted an analysis in 2015 on the long-term outcomes of patients with calcium-containing stones over a 25-year period. However, only 1.6% of their patients were on stone prevention MT, allowing them to study the natural progression of kidney stone disease. By contrast, our study was designed to explore the impact of MT on long-term recurrence of calcium stones. Consequently, owing to differences in patient characteristics and study design, our study significantly differs from the aforementioned studies. Besides, our patient cohort was at higher risk of recurrence from the outset because of history of stones in most patients.

A recent randomized controlled trial examined the longitudinal outcomes of calcium-containing kidney stones, but the median follow-up was only 2.9 years.^14^ A longer follow-up duration is particularly important because, in our study, the effect of MT was only evident after 5 years of being on it. Hence, follow-up duration should be considered when planning future studies on stone prevention.

The average follow-up of our patients after enrollment was 10.5 years. We found a higher risk of recurrence among patients who had a history of stone events, with those with higher number of events being at a proportionally higher risk.

Our study shows that being on MT is associated with 56.4% lower risk of stone recurrence among patients with a history of calcium-containing kidney stones. Previous studies have also shown benefit of drug interventions in kidney stone prevention.^15–19^ Thiazides reduce urinary calcium by increasing passive calcium reabsorption in the proximal tubule.^20,21^ Citrate acts as an inhibitor of stone crystallization and growth and hence is useful in patients with hypocitraturia.^22^ Allopurinol has been shown to prevent kidney stones in patients with high urine uric acid but normal urine calcium.^23,24^ A recent randomized control trial^14^ comparing placebo with varying doses of HCTZ, however, did not find any significant difference in symptomatic or radiographic recurrence of calcium-containing kidney stones over a 2.9-year median follow-up. Interestingly, the authors excluded patients who were receiving any kind of alkali therapy, including potassium citrate. The authors noted that patients on thiazides had lower urinary citrate over the study period compared with placebo, although none of the included patients were started on potassium citrate. In addition, as seen from our study, the benefit of the MT is not evident until at least after 5 years of treatment; it is quite possible that a 2.9-year follow-up period may not have been sufficient to see a beneficial effect. Krambeck and colleagues studied the recurrence of brushite stones and reported recurrence after a mean of 33 months.^6^ A previous randomized control trial examined the effect of twice daily HCTZ on stone recurrence and found it to be significantly effective in stone prevention.^19^ It is possible that the type and frequency of thiazide used can affect stone recurrence as well.

We found that having a stone subtype of ≥51% CaP was associated with significantly higher risk of recurrence (94% higher risk than ≤50% CaP subtype). This is consistent with the only significant finding in 24-hour urine analysis, namely urine pH > 6, associated with 109% higher risk of recurrence. Previous studies have reported higher recurrence for brushite form of calcium stones.^6,13^ Pak and colleagues previously showed that CaP subtype was more commonly associated with hypercalciuria and renal tubular acidosis.^25^ Twenty-four-hour urine studies also show high urine pH and low urinary citrate.^6^ Incomplete distal renal tubular acidosis is also a possibility, which is often underdiagnosed, primarily due to lack of standardized diagnostic tests available.^26^

In the multivariate analysis, urine pH > 6 was identified as an independent risk factor for the recurrence of kidney stones, regardless of the higher CaP mineral content. It is important to note that the average urine pH for our study group as a whole was relatively high at 5.97±0.51. This was likely due to excluding patients with uric acid stones, who typically have lower urine pH. Therefore, the clinical significance of this discovery should be interpreted with caution.

The recurrence rate in our study was the highest among patients who had ≥51% CaP subtype and were not on MT. The risk declined with MT, across all subtypes. Other than urine pH > 6, there was no other significant difference in 24-hour urine analysis between patients who had stone recurrence versus those who did not. The utility of 24-hour urine analysis for stone prevention has been debated in past^27^ and has led some others to propose empiric therapy for kidney stones based on stone composition with pharmacotherapy with thiazides and potassium citrate.^28^ Our study clearly demonstrates that among patients with pure calcium stones who have had prior stone episodes and are at high risk of recurrence at baseline, 24-hour urine study is difficult to obtain and does not impact future recurrence beyond that influenced by stone subtype of ≥51% CaP. On the other hand, MT is associated with reduction of the risk of recurrence by 48%, across all stone subtypes, and is still underutilized.

Our study suggests that the absence of initial 24-hour urine results should not dissuade clinicians from prescribing empiric MT. However, it is important to note that our study only analyzed the 24-hour urine test conducted at baseline and not follow-up 24-hour urine because of paucity of number. Further research is needed to fully understand the role of 24-hour urine tests in managing kidney stones but it also highlights the difficulty in obtaining consistent 24-hour urine studies.

Our study has some limitations. It is retrospective study relying on data collection, especially with regard to compliance with MT. However, our chart review was thorough, and we confirmed medication adherence *via* various methods (medication reconciliation data, as well as review of individual office visit notes where a specific template was used to query medication adherence as a part of the multidisciplinary stone clinic template). Second, the adequacy of 24-hour urine collection could not be confirmed because most patients had only one 24-hour collection.^29^ Finally, our study was conducted in a single academic center. We are a large tertiary care hospital with a specialized multidisciplinary joint nephrology-urology clinic specifically caring for patients with complex kidney stone disease. However, this clinic was founded in 2015, a few years after the initial enrollment of the patients in this study, and in all likelihood, did not affect the outcome of this study. Overall, the stone characterization and 10- to 12-year follow-up are unique strengths of this study. Our outcomes are also well defined and granular. Patients with only pure metabolic calcium-containing stones were included. While there are studies with a follow-up time of up to 5 years, these limited studies were performed over 30 years ago and may not represent our current patient population or physician-prescribing practices.^17,30^

In conclusion, long-term MT is an effective strategy for preventing future stone episodes among patients who have experienced at least one calcium-containing stone event, particularly for patients with ≥51% CaP subtype. Researchers should consider the fact that MT can take at least 5 years to demonstrate a significant impact, when planning future studies.

## Data Availability

All data are included in the manuscript and/or supporting information.
